# Distinguishing features in the presentations of childhood inflammatory brain diseases at a tertiary-care centre

**DOI:** 10.1186/1546-0096-10-S1-A81

**Published:** 2012-07-13

**Authors:** Tania Cellucci, Pascal N Tyrrell, Marinka Twilt, Gordon S Soon, Ivanna Yau, Shehla Sheikh, Susanne M Benseler

**Affiliations:** 1 The Hospital for Sick Children, Toronto, ON, Canada

## Purpose

The spectrum of childhood inflammatory brain diseases (IBrainD) has expanded rapidly over the past decade. Early diagnosis and treatment are critical to prevent brain damage in many disorders. Making the diagnosis is especially challenging due to overlapping clinical presentations and the presenting features of different IBrainD have not been systematically compared in children. The aim of this study was to characterize and compare the clinical, laboratory, and imaging features at presentation for the different childhood IBrainD at our institution.

## Methods

A single centre cohort study was performed with consecutive children up to 18 years of age who were diagnosed with IBrainD from June 1989 to December 2010. Patients who were seen at The Hospital for Sick Children were identified by a search of the BrainWorks database. Demographic, clinical, laboratory, neuroimaging and histologic data from the time of diagnosis were collected. Data were analysed using descriptive and univariate statistical analysis. Cluster analysis will also be performed.

## Results

A total of 165 children (47% females, median age 8.3 years) with IBrainD were identified: 124 primary angiitis of the central nervous system (cPACNS), 11 secondary CNS vasculitis (due to systemic lupus erythematosus in 4, systemic vasculitis in 2, Crohn’s disease in 1, and infection in 4), 6 neuronal-antibody associated syndromes, 6 post-infectious IBrainD, 4 other diagnoses (2 neurosarcoidosis, 1 channelopathy, 1 moya moya), and 14 non-specific IBrainD. Male sex (71%) and motor symptoms (87%), such as hemiparesis and ataxia, were more prominent in children with angiography-positive cPACNS compared to other IBrainD. Children with angiography-negative cPACNS (AN-cPACNS) and neuronal-antibody associated syndromes were more likely to be female (74% and 83%, respectively) and present with behaviour changes (56% and 50%) and cognitive dysfunction (35% and 50%) compared to other conditions. These two diagnoses could be further distinguished by the presence of headache in 62%, seizures in 79%, and abnormal MRI findings in 91% of patients with AN-cPACNS, compared to 17%, 33%, and 17%, respectively, in patients with neuronal-antibody associated syndromes.

## Conclusion

Differences observed in groups of symptoms (motor vs. cognitive-behaviour) at presentation should be considered in the development of distinct diagnostic criteria for the various childhood IBrainD, leading to earlier diagnosis and appropriate treatment.

## Disclosure

Tania Cellucci: None; Pascal N. Tyrrell: None; Marinka Twilt: None; Gordon S. Soon: None; Ivanna Yau: None; Shehla Sheikh: None; Susanne M. Benseler: None.

